# High-level intracellular expression of heterologous proteins in *Brevibacillus choshinensis* SP3 under the control of a xylose inducible promoter

**DOI:** 10.1186/1475-2859-12-12

**Published:** 2013-02-01

**Authors:** Nunzia D’Urzo, Manuele Martinelli, Chiara Nenci, Cecilia Brettoni, John L Telford, Domenico Maione

**Affiliations:** 1 Novartis Vaccines and Diagnostics, Via Fiorentina 1, 53100, Siena, Italy

**Keywords:** *Brevibacillus*, Xylose inducible promoter, Xylose uptake, Green fluorescent protein, TcdA-GT, Recombinant protein expression, Gram positive bacteria

## Abstract

**Background:**

In past years research has focused on the development of alternative Gram positive bacterial expression systems to produce industrially relevant proteins. *Brevibacillus choshinensis* is an easy to handle non-sporulating bacterium, lacking extracellular proteases, that has been already shown to provide a high level of recombinant protein expression. One major drawback, limiting the applicability of the *Brevibacillus* expression system, is the absence of expression vectors based on inducible promoters. Here we used the *PxylA* inducible promoter, commonly employed in other *Bacillae* expression systems, in *Brevibacillus*.

**Results:**

Using GFP, α-amylase and TcdA-GT as model proteins, high level of intracellular protein expression (up to 250 mg/L for the GFP) was achieved *in Brevibacillus*, using the pHis1522 vector carrying the *B. megaterium* xylose-inducible promoter (*PxylA*)*.* The GFP expression yields were more than 25 fold higher than those reported for *B. megaterium* carrying the same vector. All the tested proteins show significant increment in their expression levels (2-10 folds) than those obtained using the available plasmids based on the P2 constitutive promoter.

**Conclusion:**

Combining the components of two different commercially available Gram positive expression systems, such as *Brevibacillus* (from Takara Bio) and *B. megaterium* (from Mobitec), we demonstrate that vectors based on the *B. megaterium PxylA* xylose inducible promoter can be successfully used to induce high level of intracellular expression of heterologous proteins in *Brevibacillus*.

## Background

Bacterial expression systems for heterologous protein production are attractive because of the ability of the host cell to grow rapidly and at high density on inexpensive substrates. *Escherichia coli* is the most extensively used bacterial host for production of recombinant proteins. In spite of its large employment, *E. coli h*as several disadvantages that limit its applicability in the industrial production of proteins: 1) The expression of recombinant proteins often results in their accumulation as insoluble aggregates in inclusion bodies; 2) The separation of lipopolysaccharide (LPS), generally referred as pyrogenic endotoxins, requires the introduction of additional expensive purification steps [1]. Therefore in past years, research has focused on the development of an alternative bacterial expression system. Gram positive bacteria do not produce LPS and are able to export proteins directly into the extracellular medium. Several expression systems for heterologous protein production in Gram positive bacteria, especially *Bacilli*, have already been successfully used to produce a number of industrial relevant proteins [2,3]. Recently Takara Bio has released a *Brevibacillus choshinensis* (previously known as *Bacillus brevis*) expression system that is reported to guarantee high levels of extra cellular protein expression [4] although few data are available about the performance of the intracellular expression [5]. Although less characterized than *B. subtilis* and *B. megaterium, Brevibacillus choshinensis* HPD31-SP3 (*Brevibacillus*) presents attributes that make it an advantageous host for heterologous protein production. A simple protocol to induce competence for DNA uptake up to 10^4^cfu/μg without protoplast preparation is available from Takara Bio. *Brevibacillus* readily grows up to high cell density in shake flasks using either complex or chemically defined media suitable for uniform ^15^N isotopic protein labelling [6]. Moreover, similarly to *B. megaterium*, the commercially available *Brevibacillus* strain does not form spores and presents limited intracellular and extracellular protease activities. All these aspects together make this bacterium attractive because it is more user-friendly compared to other Gram positive microorganisms. A clear disadvantage though is that all the commercially available expression vectors designed for *Brevibacillus choshinensis* SP3 rely on constitutive promoters and therefore do not allow a tightly controlled gene expression. This poses a limit to the expression of foreign proteins potentially toxic to the host cell and can impact on the final yield because of a sustained metabolic burden.

Efficient sugar-inducible promoters such as *Plac*, *Pbad, *and *rhaPbad* of *E. coli*[7-9] and *Pxyl* of *Bacillus subtilis*[10] are widely used to optimize gene expression in the respective micro-organisms. For the *Bacillae*, one of the most commonly employed expression system is based on the *PxylA* inducible promoter of the *xyl* operon, which is usually localized on a freely replicating plasmid containing a copy of the *xylR* gene along with it [11]. Repression of promoter activity in the absence of the inducer xylose is mediated by the repressor protein XylR. The selection of microbial hosts able to uptake xylose but deficient in its utilization is essential to maintain the concentration of the inducer constant during cultivation and achieve high expression yields [3]. D-xylose metabolism in bacteria typically involves transport, isomerization to D-xylulose, and phosphorylation to D-xylulose-5-phosphate. The second and third steps of this pathway are catalyzed by D-xylose (D-glucose) isomerase (XylA) and xylulose kinase (XylB), respectively [12], while xylose uptake is mediated via at least two distinct transport systems: one involves a D-xylose–H^+^ or –Na^+^ symporter (XylE/XylT) [13,14]. The second mechanism consists of genes for xylose ABC transport form a *xylFGHR* operon in which *xylF*, *xylG*, and *xylH* code for the periplasmic xylose-binding protein, ATPase, and permease, respectively [15].

No *xylA* homologues were found in the *Brevibacillus brevis NBRC 100599* genome supporting that, as already reported, *Brevibacillus* species do not ferment D-xylose [16]*.* However, the outcome of a xylose induction system in *Brevibacillus* is not obvious because no homologues of the XylE/XylT and XylFGH systems, involved in D-xylose uptake in other organisms [17], are present in the *Brevibacillus* genome.

To confirm the suitability of a *PxylA* promoter in *Brevibacillus*, we compared the intra/extra-cellular expression level of the green fluorescent protein (GFP) [18], α-amylase from *B. licheniformis*[19] and the Y283A-D285A-D287A mutant of TcdA catalytic domain from *Clostridium difficile* (TcdA-GT) [5], using the xylose-inducible promoter *PxylA* and the strong constitutive promoter P2. Here we show that the *PxylA* xylose inducible promoter is able to induce high levels of intracellular expression of heterologous protein in *Brevibacillus*.

## Results

### Sequence Search

The *Brevibacillus* genome was analyzed to find homologues of the proteins known to be involved in xylose uptake and metabolism in other organisms and therefore to evaluate its suitability as a host for protein expression using the xylose induction. A PSI-BLAST search (E <0,1) was performed to detect putative xylose import systems in the *Brevibacillus brevis NBRC 100599* genome. No homologues with high a degree of identity to the XylA, XylE and XylF genes were found. When using the *E. coli* XylA (GenBank: NP_418022) as input, no putative homologues were found in the *Brevibacillus brevis NBRC 100599* genome. The BLAST search using the *E. coli* XylE (GenBank: NP_418455) as input could only identify an alpha-ketoglutarate permease (YP_002770906) with low sequence identity with *E. coli* XylE (20% identity) and *B. megaterium* XylT (18,5% identity). The BLAST search using *E. coli* XylF (GenBank: AAB18543) as input revealed two putative ABC transporter substrate binding proteins (mglb and BBR47_06790). The mglb protein showed 23,8% identity with XylF and the absence of 3 of the 5 xylose binding residues [20], (D135, N137, K242). The BBR47_06790 showed 17,8% identity when compared to XylF and once again the absence of 3 of the 5 xylose binding residue D135, N196, K242.

### *Brevibacillus* growth profile in complex and minimal media

First, we determined the rate of growth of *Brevibacillus* in shaking flask containing TM medium and incubated at different temperatures (Additional file 1: Figure S1). A final OD_600_ of about 5 was obtained growing *Brevibacillus* in a temperature range between 25°C and 37°C, while no significant growth was observed at 20°C after 120 h. To confirm the inability to metabolize xylose, *Brevibacillus* was cultured in minimal medium with either glucose or xylose as unique carbon source. After 24 h of culture at 37°C, 150 rpm in shaking flask, no growth (OD_600_ <0,1) was observed for *Brevibacillus* using chemically defined medium containing xylose while an OD_600_ of about 1 was detected for *Brevibacillus* grown in the same media containing glucose.

### Intracellular GFP expression in *Brevibacillus*

**Figure 1:**
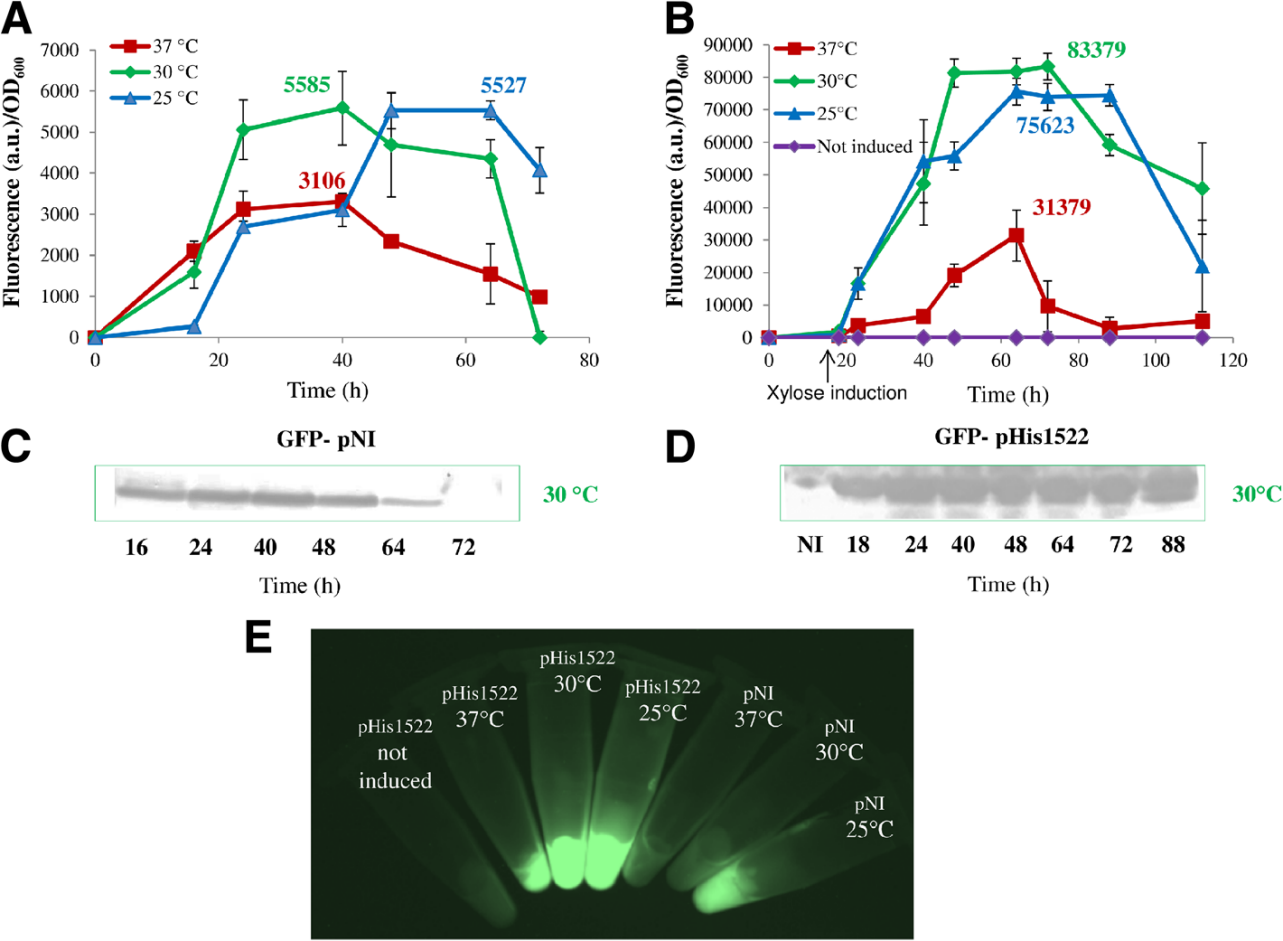
**Time course of intracellular GFP production by *Brevibacillus choshinensis *SP3 at different temperatures. A**) GFP fluorescence of *Brevibacillus *cells carrying GFP-pNI grown at 37°C (red line), 30°C (green line), 25°C (blue line) **B**) GFP fluorescence of *Brevibacillus *cells carrying the GFP-pHis1522 grown at 37°C (red line), 30°C (green line), 25°C (blue line) **C**) Western blot analysis of *Brevibacillus *cells carrying GFP-pNI grown at 30°C **D**) Western blot of *Brevibacillus *cells carrying GFP-pHis1522 grown at 30°C **E**) Fluorescence imagine of *Brevibacillus *cells carrying GFP-pNI and GFP-pHis1522 after 48 h of growth at 37°C, 30°C, 25°C (about 20 OD_600_). The GFP-pHis1522 was induced with 0,5% of xylose. 5 mL of culture were centrifuged and the image was carried out from the cell pellet using ImageQuant 400 (GE Healthcare). All cultures were grown in triplicate, and each experiment was performed at least twice. Error bars indicate standard deviations.

GFP production by *Brevibacillus* carrying the GFP-pHis1522 induced with different amounts of xylose was compared to cultures transformed with the GFP-pNI vector. The influence of temperature on protein yield was assessed for all clones by western blot analysis and fluorescence intensity determination (Figure 1). The GFP-pNI *Brevibacillus* clone showed a maximum of total GFP production at 30°C after about 40 h (5585 Fluorescence Unit (F.U.) /OD_600_) (Figure 1A). Different incubation temperatures affected the rate of synthesis of GFP (Figure 1A) also if comparable final yields of total protein were obtained at 25°C and 30°C. The GFP-pHis1522 *Brevibacillus* clone showed maximum GFP production at 30°C about 48 h after induction with 0,5% of xylose (83379 F.U./OD_600_) (Figure 1B). The xylose concentration slightly affects both the rate of protein expression and total protein yield (Additional file 2: Figure S2A) confirming the inability of *Brevibacillus* to metabolize the xylose. In this range of temperatures, the GFP expression levels, induced by xylose, are about 10 fold higher with respect those achievable with the GFP-pNI (Figure 1). No significant basal expression of GFP was detected growing *Brevibacillus* carrying GFP-pHis1522 for 96 h without induction, confirming that protein expression is repressed in absence of xylose (Figure 1). At 37°C the performance of both inducible and non inducible vectors were drastically reduced.

### Intracellular α-amylase expression in *Brevibacillus*

**Figure 2:**
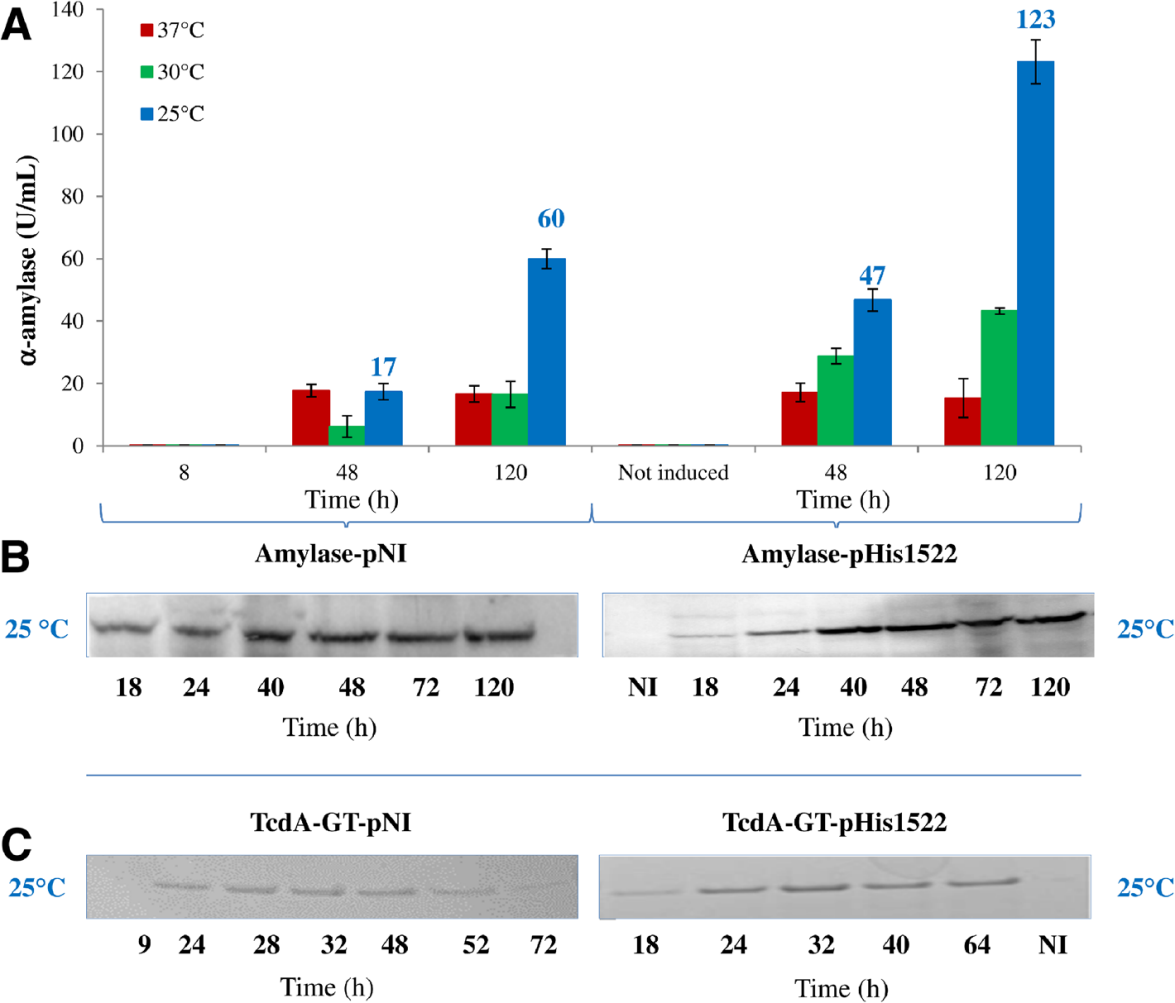
**Time course of intracellular α-amylase and TcdA-GT production by *Brevibacillus choshinensis *SP3 at different temperatures. A**) α-amylase activity of *Brevibacillus *lysate carrying α-amylase-pNI and α-amylase-pHis1522 grown in at 37°C (red line), 30°C (green line), 25°C (blue line) **B**) Western blot of *Brevibacillus *cells carrying α-amylase-pNI and α-amylase-pHis1522 grown at 25°C **C)** Western blot of *Brevibacillus *cells carrying TcdA-GT-pNI and TcdA-GT-pHis1522 grown at 25°C α-amylase-pHis1522 and TcdA-GT-pHis1522 were induced with 0,5% of xylose. All cultures were grown in triplicate, and each experiment was performed at least twice. Error bars indicate standard deviations.

α-amylase production by *Brevibacillus* carrying the α-amylase-pHis1522 induced with 0,5% of xylose was compared to those obtained with the α-amylase-pNI vector. The influence of temperature on protein expression was assessed for all clones by western blot analysis and by measuring the amylase activity after 48 and 120 h of induction. Both α-amylase-pNI and α-amylase-pHis1522 *Brevibacillus* clones showed a maximum of total protein production at 25°C after about 120 h of culture (60 U/mL and 123 U/mL respectively) (Figure 2) while at 37°C and 30°C the performance of both inducible and non inducible vectors were reduced. Interestingly the expression in pHis1522 vector was more than 2 fold higher compared to those achievable with the GFP-pNI vector (Figure 2). No significant basal expression of α-amylase was detected growing *Brevibacillus* carrying GFP-pHis1522 for 120 h without induction, confirming that protein expression is repressed in absence of xylose (Figure 2A and 2B).

### Intracellular TcdA-GT expression in *Brevibacillus*

TcdA-GT production by *Brevibacillus* carrying the TcdA-GT-pHis1522 induced with 0,5% of xylose was compared to those obtained with the α-amylase-pNI vector. The influence of temperature on protein yield was assessed for all clones by performing SDS-PAGE analysis and BCA quantification of the IMAC purified samples (Figure 2C). In both TcdA-GT-pNI and TcdA-GT-pHis1522 *Brevibacillus* clones, the best results in terms of protein expression and stability were observed at 25°C, while lower expression levels were detected using both vectors at 37°C and 30°C as already observed for α-amylase (data not shown).

### Extracellular GFP expression in *Brevibacillus*

**Figure 3:**
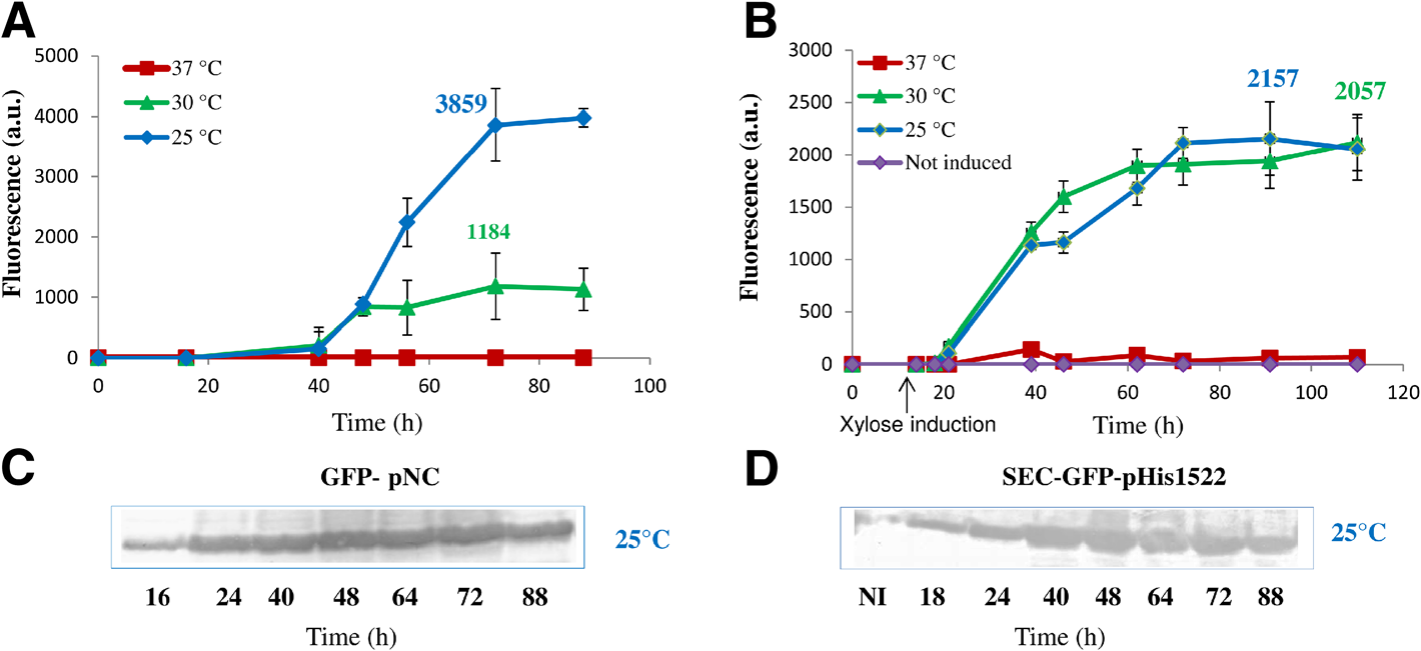
**Time course of extracellular GFP production by *Brevibacillus choshinensis *SP3 at different temperatures. A**) GFP fluorescence of culture supernatant of *Brevibacillus *carrying GFP-pNC grown at 37°C (red line), 30°C (green line), 25°C (blue line) **B**) GFP fluorescence of culture supernatant of *Brevibacillus *carrying the SEC-GFP-pHis1522 grown at 37°C (red line), 30°C (green line), 25°C (blue line) **C**) Western blot of the culture supernatant of *Brevibacillus *carrying GFP-pNC grown at 25°C **D**) Western blot of the culture supernatant of *Brevibacillus *carrying SEC-GFP-pHis1522 grown at 25°C. SEC-GFP-pHis1522 was induced with 0,5% xylose. All cultures were grown in triplicate, and each experiment was performed at least twice. Error bars indicate standard deviations.

GFP production by *Brevibacillus* carrying the SEC-GFP-pHis1522 induced with different amounts of xylose was compared to those obtained with the GFP-pNC vector. The influence of temperature on protein yield was assessed for all clones by western blot analysis and fluorescence intensity determination (Figure 3). As already observed for intracellular expression, GFP-pNC and SEC-GFP-pHis1522, showed the best performance at 25-30°C (Figure 3A and 3B). The GFP-pNC *Brevibacillus* clone showed the peak of fluorescence in the supernatant after 72 h of growth (3859 F.U.) (Figure 3A and 3C); the SEC-GFP-pHis1522 *Brevibacillus* clone showed maximum GFP production after about 74 h of induction with 0,5% of xylose (2157 F.U.) (Figure 3B and 3D). As already observed for the intracellular expression, the xylose concentration had a minor effect on the total protein yield (Additional file 2: Figure S2B) and no significant basal expression was detected in SEC-GFP-pHis1522 *Brevibacillus* clone (Figure 3B). At 25°C and 30°C the maximum expression levels, achievable with the *PxylA* promoter, were similar to those obtained with the P2 promoter (Figure 3) and significantly lower than those achievable with the intracellular expression. No detectable GFP production was observed at 37°C with either of the two plasmids.

### Extracellular α-amylase expression in *Brevibacillus*

**Figure 4:**
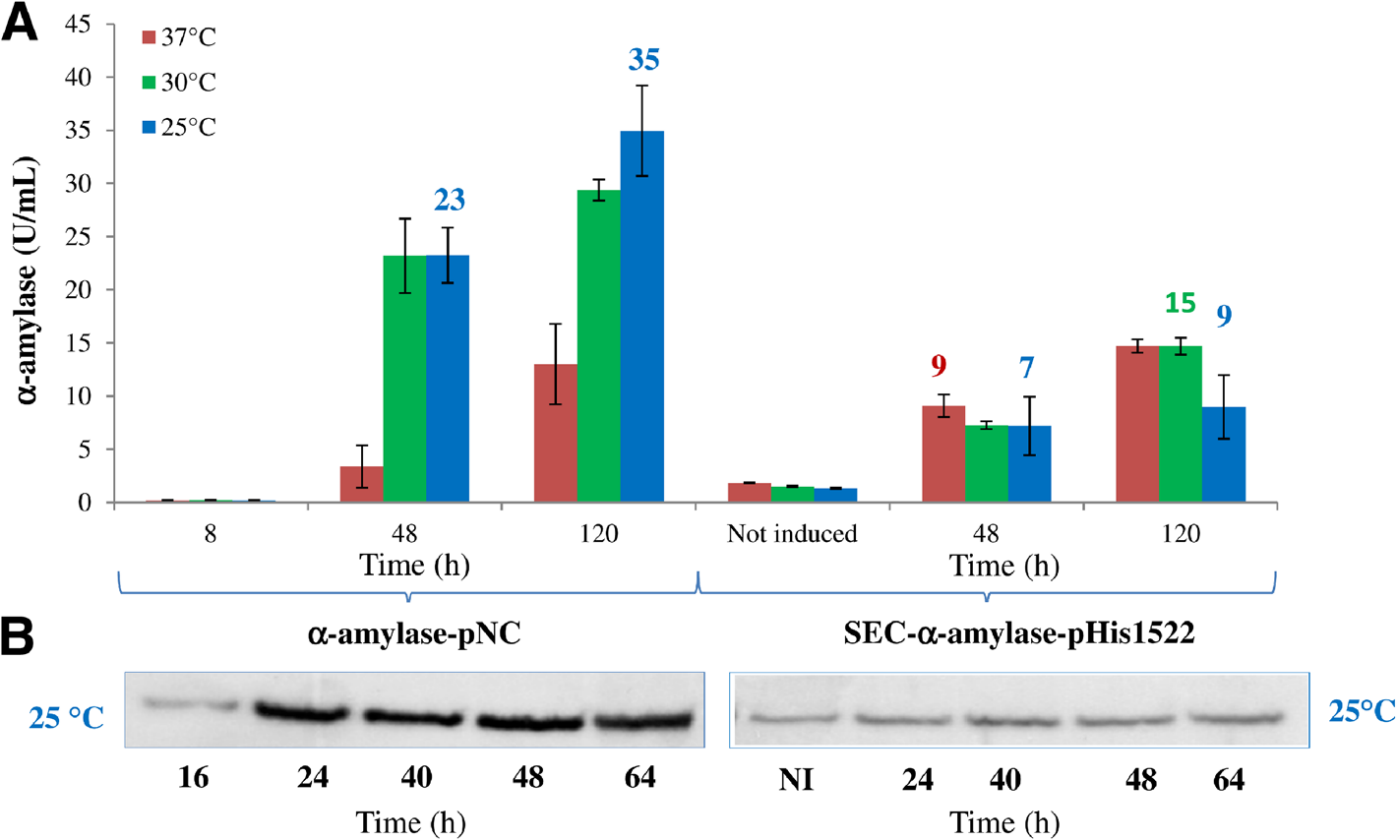
**Time course of extracellular α-amylase production by *Brevibacillus choshinensis *SP3 at different temperatures. A**) α-amylase activity of culture supernatant of *Brevibacillus *carrying α-amylase-pNC and SEC-α-amylase-pHis1522 grown at 37°C (red line), 30°C (green line), 25°C (blue line) **B**) Western blot of the of culture supernatant of *Brevibacillus *carrying α-amylase-pNC and SEC-α-amylase-pHis1522 grown at 25°C.The α-amylase-pHis1522 was induced with 0,5% of xylose. All cultures were grown in triplicate, and each experiment was performed at least twice. Error bars indicate standard deviations.

**Figure 5:**
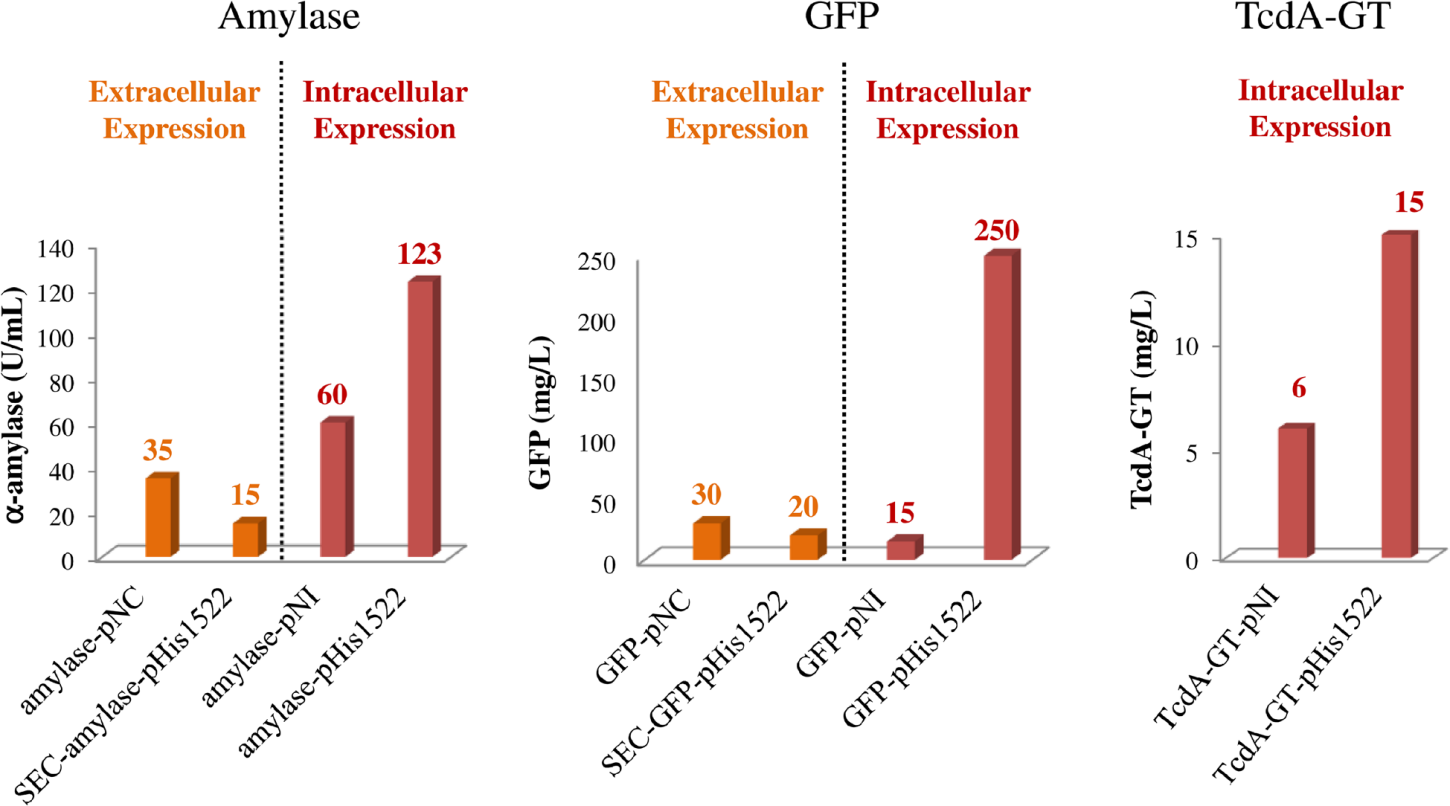
**GFP, α-amylase, and TcdA-GT production yield in *Brevibacillus SP3*. **Comparison of the maximum yield obtained for each protein/vector combination used in this study. All cultures were grown in triplicate and the reported values correspond to the average.

α-amylase production by *Brevibacillus* carrying the SEC-α-amylase-pHis1522 induced with 0,5% of xylose was compared to those obtained with the α-amylase-pNC vector. The influence of temperature on protein yield was assessed for all clones by western blot analysis and by measuring the amylase activity at 48 and 120 h of induction (Figure 4). The α-amylase-pNC *Brevibacillus* clone showed a maximum of total protein production at 25°C after about 120 h of culture (35 U/mL) (Figure 4A) while at 37°C and 30°C the performances were reduced. The expression level achievable with the *PxylA* promoter was not affected by the temperature and, in all tested conditions, was lower than that obtained with the P2 promoter (Figure 5).

### Purification of the recombinant GFP and TcdA-GT

To quantify the amount of recombinant GFP and TcdA-GT produced by different expression vectors, 50 mL of each culture was collected at the peak of fluorescence, purified by IMAC chromatography and the amount of purified protein was estimated by BCA assay. The result confirms that pHis1522 induces higher level of GFP and TcdA-GT intracellular protein expression compared to the pNI-His vector. However, no improvement of extracellular protein production was observed using pHis1522 in comparison to pNC-His vector, using the same secretion signal peptide. In fact, the GFP-pHis1522 clone produced the highest protein yield (250 mg/L) (Figure 5) with roughly a 10 fold higher yield compared to all the other tested clones and TcdA-GT-pHis1522 clone produced about 3 fold higher yield than TcdA-GT-pNI clone (15 mg/L vs 6 mg/L) (Figure 5). In contrast, the yield achieved using the SEC-GFP-pHis1522 (20 mg/L) was slightly lower than that obtained when using the GFP-pNC vector (30 mg/L). These values are in agreement with the relative amount of GFP predicted using the fluorescence assay.

## Discussion

In recent years, various studies have revealed that non-pathogenic Gram positive *Bacillae* are attractive hosts for the expression of heterologous proteins. They show in fact several favourable features, such as the natural capacity to secrete which allows the exporting of proteins directly into the extracellular medium, and the absence of endotoxins which make it highly attractive for the industrial production of pharmaceutical proteins. Several factors including (1) lack of suitable expression vectors, (2) plasmid instability, (3) presence of proteases, (4) lack of spore deficient strains, and (5) low transformation efficiency, limit the choice of expression systems based on *Bacillae* with respect to *E. coli.* Recently, Takara Bio has released a *Brevibacillus* Expression System that overcomes the majority of these limitations by combining an easy to handle expression host with the most favorable features typical of Gram positive bacteria. A number of different proteins such as bacterial α-amylase [21], cholera toxin B subunit B [22], human epidermal growth factor [23] and human interleukin-2 [24] have already been successfully secreted in high amounts by using this system. Moreover, the intracellular expression, though less explored, has recently permitted the production of GT-domain of clostridial TcdA [5]. In this work for the first time, we successfully employ the *PxylA* inducible promoter of *B. megaterium* to produce heterologous proteins in *Brevibacillus*. The selection of an expression host able to import the xylose into the cells but deficient in its utilization is essential to maintain a constant level of inducer during the culture and to achieve a high efficiency of target gene induction. A BLAST search across the *Brevibacillus brevis NBRC 100599* genome did not reveal any XylA and XylB homologues. These findings, coupled with the inability of *Brevibacillus* to grow in minimal medium with xylose as the sole carbon source, confirm that, differently from *B. megaterium*[25], the *Brevibacillus* strain used in this work is naturally deficient in xylose utilization. In agreement with this, the use of pHis1522 vector, carrying the xylose inducible promoter in *Brevibacillus*, allow a high level of intracellular protein expression. The yield obtained for GFP (250 mg/L) is more than 25 fold higher than that reported for *B. megaterium* carrying the same expression vector [26], more than 10-fold higher compared to that obtained with *Brevibacillus* carrying the GFP-pNI vector (Figure 5) based on the P2 constitutive promoter and, finally, comparable with *E. coli* in a fed-batch cultivation [27]. The yields obtained for both α-amylase and TcdA-GT using the pHis1522 vector were about 2-3 fold higher compared to those obtained using the pNI-His vector. In addition "non-induced cultures" (Figures 1, 2, 3 and 4) confirmed that protein expression is well repressed in absence of xylose. The protein induction was maintained for long term after the addiction of xylose (up to 60-120 h) and the expression level was not affected by the xylose concentration. Consequently *Brevibacillus* is deficient in the xylose utilization but is also able to uptake the sugar into the cells even in absence of the representative genes required for xylose transport. Further investigations will be necessary to understand if an alternative uncharacterized xylose import system is present in *Brevibacillus* or whether the uptake of xylose could occur via a transporter with broad substrate specificity, as already observed with *B. subtilis*[28]. On the other hand, the levels of secreted proteins obtained using both the pNC-His and pHis1522 vectors were lower than expected and comparable to those obtained with pNI-His. While no published data are available regarding the GFP production using *Brevibacillus*, a high level of α-amylase production was already reported using the same expression vector [29]. The low yields obtained using the SEC-GFP-pHis1522 may not be due to the low efficiency of the xylose induction system that performs robustly in terms of intracellular protein expression, but rather to the saturation of the protein secretion system. Further investigation will be necessary to understand if the replacement of the *Brevibacillus* R6L2 signal peptide with others derived from *Brevibacillus* or *B. megaterium* could properly enhance the secretion ability of *Brevibacillus* in pHis1522, carrying the *PxylA B. megaterium* promoter. However, the good performance that the xylose inducible promoter showed in terms of intracellular protein expression, the absence of LPS and the simple lysis method make *Brevibacillus* attractive, in particular for all those proteins that, similarly to TcdA-GT, are poorly expressed, toxic or degraded in *E. coli*

## Conclusions

Here we demonstrated, for the first time, that the *PxylA* inducible promoter of *B. megaterium* can efficiently induce long term expression of heterologous proteins in *Brevibacillus*. Xylose induction in *Brevibacillus* produces up to 250 mg/L of intracellular GFP in shake flask cultures. The availability of controllable vectors, carrying a well repressed and regulated promoter, could largely extend the application of *Brevibacillus* in protein production. In conclusion, by shuffling the components of two different commercially available Gram positive expression systems, such as *Brevibacillus* (from Takara Bio) and *B. megaterium* (from Mobitec), we created a new original blend of potential advantage for proteins that in *E. coli* are either degraded or poorly expressed.

## Methods

### Chemicals and enzymes

α-amylase from *Bacillus licheniformis* (catalog number A-4551), anti-α-amylase antibody produced in rabbit (catalog number A8273-1VL), D-(+)-xylose (catalog number W360600), and antibiotics were purchased from Sigma Chemical Co. (St. Louis, MO, USA). The EnzChek_Ultra amylase assay kit (catalog number E33651) and the polyclonal anti-GFP Rabbit Serum (catalog number A6455) were purchased from Life Technologies Corp. (Carlsbad, CA, USA). ECL Western Blotting Detection Reagents (catalog number RPN2106), Amersham Hyperfilm ECL (catalog number 28-90-68-37) were purchased from GE Healthcare (Chalfont St. Giles, United Kingdom).

### Sequence Search

A protein BLAST search (*E* <0,1) [30] was performed in order to detect putative *E. coli* XylA, XylE, XylT and XylF homologues in the *Brevibacillus* brevis NBRC 100599 genome. A further BLAST search was performed to detect the genes codifying for putative XylG, XylH and XylR homologues and verify their respective genomic localization in *Brevibacillus*.

### Vectors modification

A new version of pNI-His, pNC-His (Takara Bio) and pHis1522 (Mobitec) vectors (respectively pNI/ccdB, pNC/ccdB and pHis1522/ccdB) were constructed to express N-term His tagged proteins and to limit the background of transformants coming from the empty vectors in the PIPE cloning. The His-TEV-CcdB-Chloramphenicol cassette was amplified from the SpeedET vector [29] using the ccdB_F and ccdB_R primers (Additional file 3: Table S1) and cloned into the multicloning sites (MCS). The pNI-His, pNC-His and pHis1522 vectors were amplified using the VpNI_F/VpNI_R primers, VpNC_F/ VpNC_R primers and VpHis1522_F/VpHis1522_R primers respectively (Additional file 3: Table S1). The unpurified insert-PCR (I-PCR) and vector-PCR (V-PCR) were mixed 1:1 (v/v) and transformed in 25 μL of *E. coli* DB3.1 competent cells (Additional file 4: Table S2).

### Gene cloning

To compare the efficiency of protein expression with the two promoters P2 (*Brevibacillus*) and *PxylA* (*B. megaterium),* the *gfp* gene of the green fluorescent protein (GFP) and the α*-amylase* gene from *B. licheniformis*[19] were introduced into pNI-His and pNC-His (Takara Bio) leading respectively to intra- and extracellular expression, and pHis1522 (Mobitec), which permits intracellular expression (Table 1 and Additional file 4: Table S2). The R6L2 signal-peptide coding region [31] was inserted into the GFP-pHis1522 vectors to build the SEC-GFP-pHis1522 (Table 1).

**Table 1:** Plasmids used in this study

**Name**	**Expression**	**Promoter**	**Antibiotic resistance**	**Tag**
**GFP-pNI**	Intracellular	P2	neomycin	N-term His-Tag
**GFP-pNC**	Extracellular	P2	neomycin	N-term His-Tag
**GFP-pHis1522**	Intracellular	*PxylA*	tetracycline	N-term His-Tag
**SEC-GFP-pHis1522**	Extracellular	*PxylA*	tetracycline	No
α**-amylase-pNI**	Intracellular	P2	neomycin	N-term His-Tag
α**-amylase-pNC**	Extracellular	P2	neomycin	No
α**-amylase-pHis1522**	Intracellular	*PxylA*	tetracycline	N-term His-Tag
**SEC-**α**-amylase-pHis1522**	Extracellular	*PxylA*	tetracycline	No
**TcdA-GT-pNI**	Intracellular	P2	neomycin	N-term His-Tag
**TcdA-GT-pHis1522**	Intracellular	*PxylA*	tetracycline	N-term His-Tag

In this study a GFP variant, called GFPmut1 or enhanced green fluorescent protein (EGFP) with amino acid exchanges in the chromophore (F64L, S65T) was used (here referred to as GFP). This double amino acid exchange results in a shift of about 100 nm in the excitation maximum to 490 nm compared to the wild-type GFP and also in a 30-fold increase of fluorescence intensity.

Moreover, to compare the intracellular expression using P2 and *PxylA* promoters, the Y283A-D285A-D287A mutant of TcdA catalytic domain from *Clostridium difficile* was amplified from TcdA-GT-pNI [5] and introduced into pHis1522 (Mobitec) (Table 1). To construct the GFP-pNI and GFP-pNC expression vectors (Table 1), the GFP [32] gene was amplified by PCR using the GFP_BamHI_F and GFP_XhoI_R primers (Additional file 3: Table S1). The PCR products were digested with BamHI/XhoI and then ligated respectively into the pNI-His and pNC-His vectors using *E. coli* TOP10 (Additional file 4: Table S2). The recombinant GFP-pHis1522, α-amylase-pNI, α-amylase-pNC, α-amylase-pHis1522 and TcdA-GT-pHis1522 expression vectors were generated by the polymerase incomplete primer extension (PIPE) method [33]. The selected genes were amplified by PCR using the F-GFP/R-GFP primers, F-amy/R-amy primers and F-tcdA/R-tcdA primers respectively, which included extensions complementary to the desired vector cloning site. The pNI/ccdB, pNC/ccdB and pHis1522/ccdB vectors were then amplified by PCR using the Vp_F/Vp_R primers (pNI-His and pNC-His vectors) and the ForpHis1522/RevpHis1522 primers (pHis1522 vector) (Additional file 3: Table S1). The unpurified I-PCR and V-PCR were mixed 1:1 (v/v) and the mixture was transformed in 25 μL of *E. coli* HK100 competent cells (Additional file 4: Table S2). The R2L6 signal-peptide [31], a modified version of the signal peptide (SP) coding region of the *Brevibacillus* cell wall protein (CWP) gene was added at the N-term of GFP- α-amylase- sequences in the pHis1522 clone to generate the SEC-GFP-pHis1522 and SEC-α-amylase-pHis1522 expression vectors [34]. The GFP-pHis1522 and α-amylase-pHis1522 vectors were amplified by PCR using the SecGFP_F/SecGFP_R and SecAmylase_F/SecAmylase_R primers, which included 19bp complementary extensions coding for the R2L6 signal peptide. A 2 μL aliquot of unpurified vector-PCR product was transformed in 25 μL of *E. coli* HK100 competent cells. The vectors were amplified in *E. coli*, extracted with a The E.Z.N.A.® Plasmid Mini Kit II (Omega Biotek, GA, USA) and their DNA sequences were confirmed by DNA sequencing.

### Growth medium composition

TM medium (1% glucose, 1% polypeptone, 0,5% meat extract, 0,2% yeast extract, 0,001% FeSO_4_ ×7H_2_O, 0,001% MnSO_4_ ×4H_2_O, and 0,0001% ZnSO_4_ ×7H_2_O) and minimal medium (0,85% Na_2_HPO_4_ ×2H_2_O, 0,3% KH_2_PO_4_, 0,3% glucose or xylose, 0,1% (NH_4_)_2_SO_4_, 0,05% NaCl; 0,01% MgSO_4_, 0,0001% Biotin, 0,0001% trace element solution) were used to grow *Brevibacillus choshinensis* SP3 (Additional file 4: Table S2).

### *Brevibacillus* growth in chemically defined medium

An pre-culture of *Brevibacillus* grown in TM medium at 30°C, 150 rpm for 16 h and then diluted 100 times in 50 mL of minimal medium containing alternatively 1,5% glucose or 1,5% xylose as unique carbon source. The bacterial cells were grown in a 250 mL shaking flask at 37°C, 150 rpm 24 h.

### Bacterial transformation

*Brevibacillus* competent cells were prepared according to the manufacturer’s instructions (Takara Bio Inc., Shiga, Japan). All the expression vectors were transformed into *Brevibacillus choshinensis* SP3 using the tris-polyethylene glycol method [4] as described by Takara Bio manual using 0,5-2 μg of total DNA. 10 μg/mL of neomycin and 20 μg/mL of tetracycline were respectively used to transform the plasmid constricts.

### Shaking-flask production of recombinant GFP, α-amylase and TcdA-GT using *Brevibacillus*

To screen for protein production freshly transformed cells were grown overnight in 10 mL of TM medium containing 10 μg/mL of neomycin (pNI-His and pNC-His vectors) or 20 μg/mL of tetracycline (pHis1522 and SEC-pHis1522 vectors) at 30°C, 150 rpm. Pre-inoculums were inoculated 1:100 in 50 mL of the same media and grown at 150 rpm at 25°C, 30°C and 37°C. Recombinant expression of the selected proteins under transcriptional control of the xylose-inducible promoter, were induced by the addition of xylose at different concentrations (0,5-1-2%) at OD_600_ about 1,5. Intracellular GFP, α-amylase and TcdA-GT samples were withdrawn at different growth phases, and subjected to Western blotting analysis, GFP fluorescence determination and α-amylase activity assay as described below.

### GFP fluorescence assay

Recombinant GFP production was monitored via fluorescence spectroscopy on a black flat bottom 96 well using a Tecan Infinite M200 reader (Tecan, Mannedorf, Switzerland). The excitation wavelength was 395 nm (bandwidth 10 nm) and the emission spectrum was recorder with gain 80 in the 420-600 nm range (bandwidth 10 nm). To eliminate background fluorescence, the intensity recorded at 480 nm was subtracted from that recorded at 510 nm.

1 OD_600_ of each bacterial culture was collected and centrifuged. To monitor the intracellular expression of GFP (for GFP-pNI and GFP-pHis1522 clones), the cell pellet was washed with 1 mL of PBS and finally resuspended in 100 μL of PBS. To reduce the fluorescence background due to the media, and reveal the secreted GFP (GFP-pNC and SEC-GFP-pHis1522 clones), the supernatant was diluted up to 2 mL with PBS buffer and subsequently concentrated (from 2 mL to 200 μL), using Amicon Ultra-0.5 10 KDa (Millipore, Billerica, USA) for 2 times. A final volume of 100 μL was finally transferred into the 96-black flat bottom well plate (Greiner Bio-One, Frickenhausen, Germany) and the fluorescence emission spectra were recorded. The GFP fluorescence (expressed as fluorescence unit = F.U.) was monitored to evaluate the relative amount of protein expressed under the different conditions tested.

### α**-**amylase quantification

To qualitatively and quantitatively asses the ability of the different expression vector to produce α-amylase in *Brevibacillus*, the α-amylase activity was measured by EnzChek amylase Assay Kit (Life Technologies Corp., Carlsbad, CA, USA). Briefly, the culture supernatant (SEC-α-amylase-pHis1522) or the *Brevibacillus* lysate (α-amylase-pHis1522) was mixed with 200 μg/mL of substrate solution (starch-BODIPY FL conjugate) and incubated for 30 minutes at 25°C. Fluorescence intensity was measured at excitation and emission wavelengths of 470 and 530 nm in an Infinite M200 spectrophotometer microplate reader (Tecan, Mannedorf, Switzerland). Analysis of culture supernatant and *Brevibacillus* lysate from the GFP-pHis1522 and SEC-GFP-pHis1522 clones were also performed as negative control.

### Western Blot analysis

To analyze the intracellular expression, the bacterial cells were suspended in CelLytic Express (Sigma-Aldrich, St. Louis, MO, USA) incubated at 25°C for 30 minutes and the cellular debris was removed by centrifugation. Soluble fractions, derived from 0,08 OD_600_ of lysed bacteria, were loaded and resolved on 4-12% NuPAGE precast gels (Life Technologies Corp. Carlsbad, CA, USA) and transferred to nitrocellulose. To analyze the extracellular expression, the bacterial cells were centrifuged and 20 μL of culture supernatant were loaded and resolved on 4-12% NuPAGE precast gels (Life Technologies Corp., Carlsbad, CA, USA) and transferred to nitrocellulose. In both cases, the membranes were probed with rabbit antibodies directed against GFP (1:100 dilution) or against α-amylase (1:1000 dilution), followed by a rabbit anti-rabbit horseradish peroxidase-conjugated secondary antibody (Dako, Glostrup, Denmark). Bands were then visualized using an Opti-4CN substrate kit (Bio-Rad Laboratories, Hercules, CA) or SuperSignal West Pico chemiluminescent substrate (Pierce, IL, USA).

Western Blot analysis were performed for all the conditions (37, 30 and 25°C) but we reported in Figures 1, 2, 3, 4 only that one providing the highest levels of protein expression, as estimated by GFP fluorescence assay or amylase activity assay Kit.

### GFP and TcdA-GT purification

GFP and TcdA-GT purification by IMAC chromatography was performed to better estimate the protein yields achievable for each strain/vector combination. For purification of intracellular GFP and TcdA-GT, cells were harvested by centrifugation, re-suspended in 10 mL of binding buffer (Tris 20 mM, NaCl 300 mM, Imidazole 10 mM, pH=8,0) and finally disrupted by sonication. Cellular debris were removed by centrifugation (8000 rpm, 4°C, 30 min). The supernatant was loaded onto a PD-10 gravity flow column (GE Healthcare, Chalfont St. Giles, United Kingdom), packed with 2 mL of Ni-NTA FF resin (Qiagen, Hilden, Germany). For purification of extracellular GFP, cells were harvested by centrifugation, culture supernatant was filtered through a 0,22 μm filter (Millipore, Billerica, USA) and loaded onto a column prepared as indicated above. 2 washing step were perfomed using 40 mL of binding buffer and 40 mL of washing buffer (Tris 20 mM, NaCl 300 mM, Imidazole 20 mM pH=8,0) and the bound protein was then eluted with the elution buffer (Tris 20 mM, NaCl 300 mM, Imidazole 300 mM pH=8,0). SDS-PAGE and BCA assay (Pierce, IL, USA) were used to check protein purity and concentration, respectively.

## Competing interests

The authors declare that they have no competing interests.

## Authors’ contributions

ND, MM and DM designed research; ND, MM, CB performed research; ND and MM analyzed data; and ND, MM, CN, JT, DM wrote the paper. All authors read and approved the final manuscript.

## Supplementary Material

Additional file 1: Figure S1 Temperature dependent growth rate of *Brevibacillus choshinensis *SP3. Shaking flask cultivation of *Brevibacillus choshinensis *SP3 carrying the pNI-His vector. All cultures were grown in triplicate, and each experiment was performed at least twice. Error bars indicate standard deviations. Click here for file

Additional file 2: Figure S2 Time course of intra- and extracellular GFP production by *Brevibacillus choshinensis *SP3 at different xylose concentration. A) GFP fluorescence of *Brevibacillus *carrying the GFP-pHis1522 grown in at 25°C induced by adding different amounts of xylose (0,5% (solid line) - 1% (dashed line) - 2% (round dots)). B) GFP fluorescence of culture supernatant of *Brevibacillus *carrying SEC-GFP-pHis1522 grown at 25°C induced by adding different amounts of xylose (0,5% (dashed line) - 1% (solid line) - 2% (round dots)). All cultures were grown in triplicate, and each experiment was performed at least twice. Error bars indicate standard deviations. Click here for file

Additional file 3: Table S1 Primers used in this study (regular: complementary overlap; bold: restriction enzyme recognition site; underlined: annealing sequence). Click here for file

Additional file 4: Table S2 Vectors, plasmids in this study. Click here for file
