# Supplementary figures and images for: High-level intracellular expression of heterologous proteins in Brevibacillus choshinensis SP3 under the control of a xylose inducible promoter
Source: Microb Cell Fact. 2013 Feb 1;12:12. doi: 10.1186/1475-2859-12-12 (PMC3582527; doi:10.1186/1475-2859-12-12)

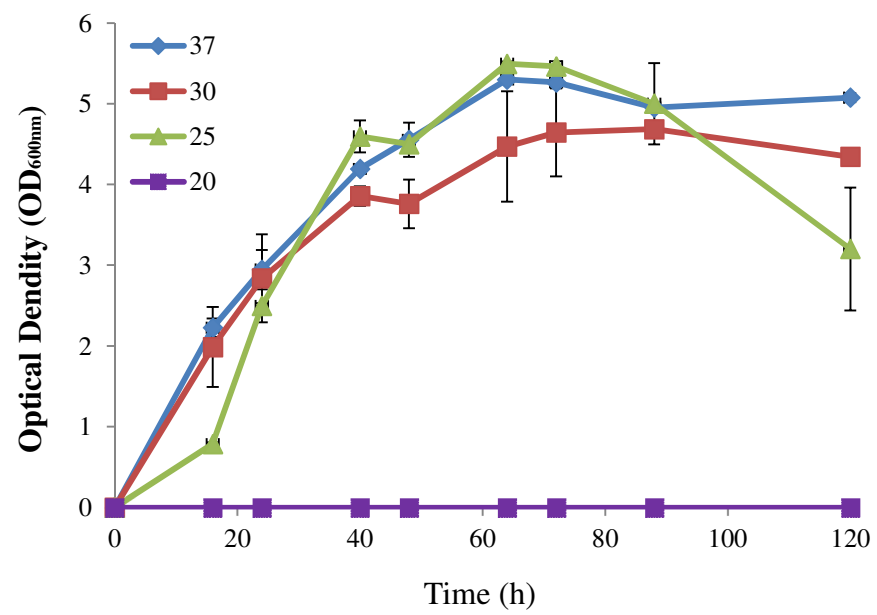

Supplement: Additional file 1: Figure S1 — Temperature dependent growth rate of Brevibacillus choshinensis SP3. Shaking flask cultivation of Brevibacillus choshinensis SP3 carrying the pNI-His vector. All cultures were grown in triplicate, and each experiment was performed at least twice. Error bars indicate standard deviations. [file 1475-2859-12-12-S1.pdf]

A

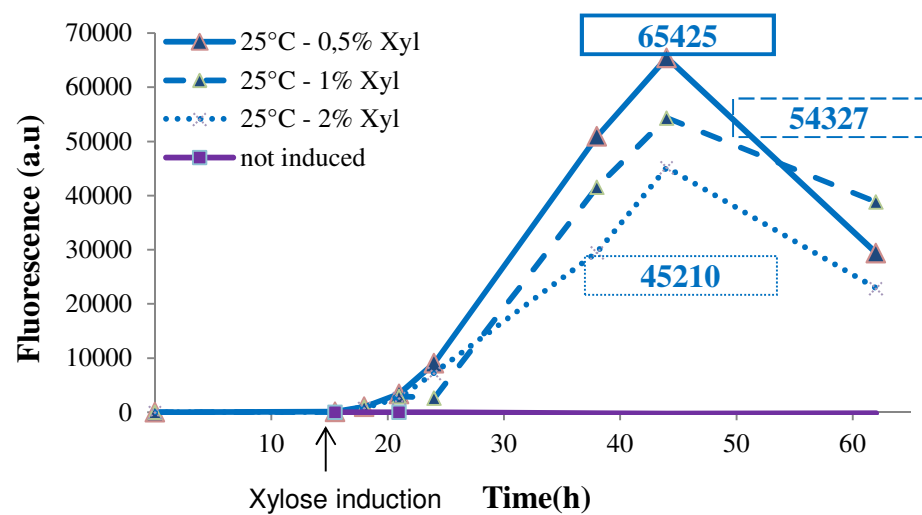

B

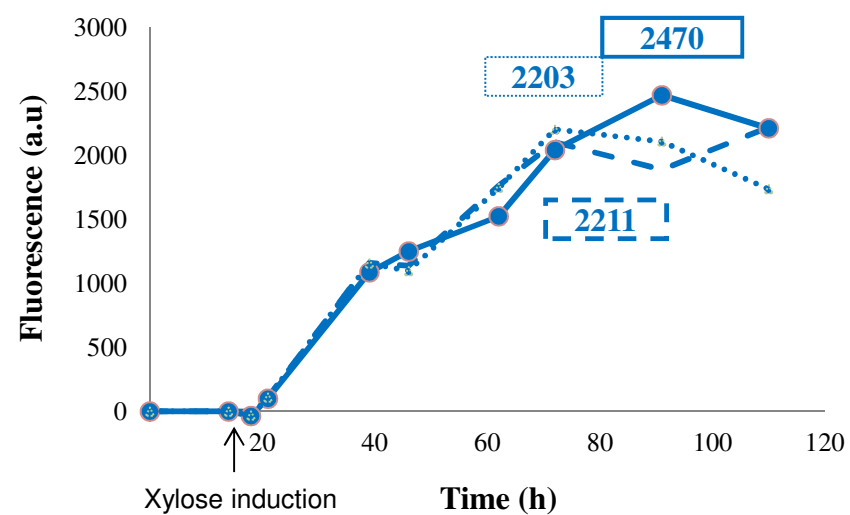

Supplement: Additional file 2: Figure S2 — Time course of intra- and extracellular GFP production by Brevibacillus choshinensis SP3 at different xylose concentration. A) GFP fluorescence of Brevibacillus carrying the GFP-pHis1522 grown in at 25°C induced by adding different amounts of xylose (0,5% (solid line) - 1% (dashed line) - 2% (round dots)). B) GFP fluorescence of culture supernatant of Brevibacillus carrying SEC-GFP-pHis1522 grown at 25°C induced by adding different amounts of xylose (0,5% (dashed line) - 1% (solid line) - 2% (round dots)). All cultures were grown in triplicate, and each experiment was performed at least twice. Error bars indicate standard deviations. [file 1475-2859-12-12-S2.pdf]
