# Supplementary material for: High-level intracellular expression of heterologous proteins in Brevibacillus choshinensis SP3 under the control of a xylose inducible promoter
Source: Microb Cell Fact. 2013 Feb 1;12:12. doi: 10.1186/1475-2859-12-12 (PMC3582527; doi:10.1186/1475-2859-12-12)
Supplement: Additional file 3: Table S1 — Primers used in this study (regular: complementary overlap; bold: restriction enzyme recognition site; underlined: annealing sequence). [file 1475-2859-12-12-S3.pdf]

**Additional file 3_Table S1: Primers used in this study (regular: complementary overlap; bold: restriction enzyme recognition site; underlined: annealing sequence).**

| **Primers** | |
|---|---|
| **Name** | **Description** |
| **GFP_BamH_F** | CGC**GGATCC**ATGGTGAGCAAGGGCGAGGAGCTG |
| **GFP_Xho1_R** | CCG**CTCGAG**TCATTACTTGTACAGCTCGTCCATGC |
| **ccdB_F** | CATCATCATCATCATCACG |
| **ccdB_R** | CTGGGGAATATAA |
| **VpNI_F** | ATGATGATGATGATGAGCCATGGCGCGTGTCAC |
| **VpNI_R** | CTGGGGAATATAACGGTACCCCGGGTTCG |
| **VpNC_F** | ATGATGATGATGATGGCTACCTGCAGCGAAAGC |
| **VpNC_R** | CTGGGGAATATAACGGTACCCCGGGTTCG |
| **VpHis1522_F** | ATGATGATGATGATGACTAGTTTGGACCATTGTAC |
| **VpHis1522_R** | GTTCTGGGGAATATAACATCACCATCACCATCAC |
| **F-GFP** | CATCATCATCATCATATGGTGAGCAAGGGCGAGG |
| **R-GFP** | ATGGACGAGCTGTACAAGTAATTATATTCCCCA |
| **ForpHis1522** | TAACGCGACTTAATTGGCCAGTGTGCCGGTCTCCG |
| **RevpHis1522** | ACTAGTTTGGACCATTGTAC |
| **SecGFP_F** | CTGCTAGCTAGTGCACTCGCACTTACTGTTGCTCCCATGGCTTTCGCTGCAGGTAGCATGGTGAGCAAGGGCGAGG |
| **SecGFP_R** | TGCACTAGCTAGCAGTAGCAGAAGCAATACACTGTTAACGACCCTTCTTTTTTTCATTGTACATTTCCCCCTTTG |
| **F-amy** | CTGTACTTCCAGGGCGCAGCAGCGGCGGCAAATC |
| **R-amy** | AATTAAGTCGCGTTATCTTTGAACATAAATTGAAACCGACCC |
| **F-tcdA** | GAAAACCTGTACTTCCAGGGCATGTCTTTAATATCTAAAGAAGAGTTAATAAAACTC |
| **R-tcdA** | AATTAAGTCGCGTTATCACTCGAGAGTGATTTTTTTGTTGAC |
| **Vp_For** | TAACGCGACTTAATTGGCCAGTGTGCCGGTCTCCG |
| **Vp_Rev** | GCCCTGGAAGTACAGGTTTTC |
| **SecAmylase_F** | CTGCTAGCTAGTGCACTCGCACTTACTGTTGCTCCCATGGCTTTCGCTGCAGCAGCGGCGGCAAATCTTAATGGG |

| | |
|---|---|
| **SecAmylase_R** | <u>CAAAGGGGGAAATGTACA</u>ATGAAAAAAAGAAGGGTCGTTAACAGTGTATTGCTTCT GCTACTGCTAGCTAGTGCA |
