# Supplementary material for: High-level intracellular expression of heterologous proteins in Brevibacillus choshinensis SP3 under the control of a xylose inducible promoter
Source: Microb Cell Fact. 2013 Feb 1;12:12. doi: 10.1186/1475-2859-12-12 (PMC3582527; doi:10.1186/1475-2859-12-12)
Supplement: Additional file 4: Table S2 — Vectors, plasmids in this study. [file 1475-2859-12-12-S4.pdf]

Additional file 4_ Table S2: Vectors, plasmids in this study.

**Strains**		
**Name**	**Description**	**Source/Reference**
B. Choshinensis SP3	Strain for recombinant protein expression	Takara Bio,Shiga, Japan
E. coli HK100	Strain for plasmid construction with PIPE cloning	[34]
E. coli TOP10	Strain for plasmid construction with ligase	Life Technologies Corp., CA, USA
E. coli DB3.1	Strain for constructing and propagating plasmids containing the ccdB gene	Life Technologies Corp., CA, USA
**Expression vectors**		
**Name**	**Description**	**Source/Reference**
**pNI-HisF**	Shuttle vector for cloning in *E. coli* and intracellular expression in *B. Choshinensis* under the control of a P2 promoter.	Takara Bio, Shiga, Japan
**pNC-HisF**	Shuttle vector for cloning in *E. coli* and protein secretion in *B. Choshinensis* under the control of a P2 promoter.	Takara Bio, Shiga, Japan
**pHis1522**	Shuttle vector for cloning in *E. coli* and intracellular expression in *B. megaterium* under the control of a *PxylA*, xylose inducible promoter.	Mobitec, Göttingen, Germany
